# Fruitful outcomes without fatal costs: non-lethal alternatives show promise in alleviating human-wildlife conflict involving an island flying fox

**DOI:** 10.7717/peerj.20859

**Published:** 2026-03-05

**Authors:** Geetika Bhanda, Ryszard Z. Oleksy, François Benjamin Vincent Florens

**Affiliations:** 1 Tropical Island Biodiversity, Ecology and Conservation Pole of Research, Faculty of Science, University of Mauritius, Le Réduit, Mauritius; 2 Ecosystem Restoration Alliance Indian Ocean, Albion, Mauritius

**Keywords:** *Pteropus niger*, Commercial fruits, Crop protection, Frugivory, Fruit bat, *Litchi chinensis*, *Mangifera indica*, Mauritius, Netting

## Abstract

Human-wildlife conflict is a growing threat to biodiversity, primarily involving damage to agricultural production. In Mauritius, the threatened Mascarene endemic Mauritian flying fox (*Pteropus niger*) has been subjected to five annual mass-culling campaigns since 2015, which failed their crop protection goals while raising the species’ extinction risk. We evaluated seven non-lethal potential deterrent methods to mitigate flying fox as well as bird damage to ripening fruits in lychee orchards. This study was conducted in close collaboration with local small- and large-scale fruit growers, who occasionally influenced the number and spatial arrangement of trees assigned to treatments and controls. We estimated expected fruit yield per tree before ripening, collected fallen fruits weekly over the four-week fruiting season and categorized them by damage agent. At five control sites, flying foxes damaged 0–88% (mean = 43%) of fruits on unprotected lychee trees. Two sites likely experienced high nocturnal human disturbance, which may have skewed flying fox damage on the control trees; excluding these sites, damage averaged 66%. The lowest flying fox damage occurred when trees were covered by netting or parallel cords (<1%), followed by a nocturnal sound-light system (4%), a nocturnal sprinkler system (11%), local traditional nocturnal smoke and lights (19%), flags positioned above the trees (25%) and flags saturated with repellent odours and placed above the trees (38%). Bird damage was less than that from flying foxes (1–12%, mean = 6%) and similar in most treatments, including netting due to holes in the nets. Testing the sound-light system in one mango orchard resulted in a reduction of flying fox damage from 60% to 20%, but an increase in bird damage from 1% to 16%. Overall, we provide evidence that various non-lethal crop protection methods are effective, although to varying degrees. In comparison, mass-culling campaigns failed to improve overall fruit production. In addition to the benefits of netting, which can carry relatively high upfront costs, this study highlights the potential value of the sound-light and sprinkler systems.

## Introduction

Human-wildlife conflict (HWC) arises when interactions between people and wild animals result in negative outcomes to either side, such as competition for resources, habitat encroachment, or direct threats to life, property, and livelihoods (Madden, 2004; Barua, Bhagwat & Jadhav, 2013; Nyhus, 2016). It is a relatively new threat to biodiversity that has grown rapidly within the last decades (Nyhus, 2016; Seoraj-Pillai & Pillay, 2016), and is expected to intensify with the continued global expansion of human activities into natural habitats (Tilman, 1999; Schmitz et al., 2014; Jaureguiberry et al., 2022; Wang, Wei & Svenning, 2023). Governance plays a critical role in shaping these interactions, yet legal aspects (such as inadequate law enforcement, weak implementation, or flawed legislation) appear to frequently exacerbate HWCs instead of promoting coexistence and species conservation (Woolaston et al., 2021). For example, the government of Kerala (India) recently declared HWC a state-specific disaster, a move that, while intended to enable rapid response, could legitimise the killing of protected wildlife as a long-term management measure (Haris, 2025). Island flying foxes (Chiroptera: *Pteropus* spp.) exemplify this challenge, being the most threatened group of bats worldwide (Kingston, Florens & Vincenot, 2023). They face numerous simultaneous threats, including hunting, persecution and habitat destruction (McConkey et al., 2004; O’Shea et al., 2016; Vincenot, Collazo & Russo, 2017; Vincenot, Florens & Kingston, 2017). The legal and illegal killing of flying foxes in response to damage to commercial fruits threatens species in Australia (*Pteropus* spp.) (Hall & Richards, 2000), Peninsular Malaysia (*P. vampyrus*) (Epstein et al., 2009), Japan (*P. dasymallus*) (Vincenot, Koyama & Russo, 2015), Mauritius (*P. niger*) (Florens, 2016) and Nicobar Islands (*P. melanotus* and *P. faunulus*) (Vincenot, Florens & Kingston, 2017).

Flying foxes fulfil key ecological and economic roles (Aziz et al., 2021) through seed dispersal (McConkey & Drake, 2015) and may have disproportionally large influence on forest structure and biomass (Florens et al., 2017a). Their ability to fly up to ∼90 km in a single night (Epstein et al., 2009), potentiates some long-distance seed dispersal (Oleksy, Racey & Jones, 2015). Although flying foxes may also disseminate invasive alien plants (Nyhagen et al., 2005), their role there remain minor compared to the many other species also disseminating alien seeds. Furthermore studies indicate that flying foxes forage predominantly in habitats where native species predominate over introduced ones (Seegobin, Oleksy & Florens, 2024) as well as prefer native species (Fall & Drezner, 2011) for which they are often the most important disseminators (Florens et al., 2017a; Albert et al., 2021; Todd et al., 2022). Flying foxes also pollinate important fruit crops (Bumrungsri et al., 2009; Nathan et al., 2009; Aziz et al., 2017) as well as native forest species (Fujita & Tuttle, 1991; Scanlon et al., 2014).

Many non-lethal approaches to protecting crops from fruit-eating bats have been devised, with several shown to be effective. The oldest and most widely documented method is netting (Korine, Izhaki & Arad, 1999; Mandelc & Carr, 2000). Netting can be installed as permanent structures, supported by frames, covering entire orchards as in Australia (Eby & Lunney, 2002), or as simpler arrangements where individual trees are enclosed by nets draped directly over them and held up with poles, as reported in Mauritius (Oleksy et al., 2021). In Madagascar, farmers use plastic flags and bells, though a taste and odour deterrent (Plantskydd^®^) sprayed on trees proved more effective (Raharimihaja et al., 2016). Artificial lights above tree canopies in India also reduced flying fox damage to some extent (Singh, Singh & Singh, 2022). However, certain barriers can reduce uptake of such methods, including access and costs of materials as well as the labour and time required for installation (Gough, 2002; Raharimihaja et al., 2016; Tollington et al., 2019). Using shotguns and smoke are also employed by orchard owners in Australia and Mauritius (Bicknell, 2002; Oleksy et al., 2021). However, the effectiveness of these methods has not been systematically assessed, leaving only anecdotal information (Ullio, 2002; Aziz et al., 2016). Effectively mitigating HWC often requires a combination of different measures (Hoare, 2012). Ultimately, optimal non-lethal approaches for reducing crop damage by flying fox are often context-dependent and site-specific, and therefore should be tested locally prior to recommendations.

Measures proven to deter flying foxes may inadvertently increase damage by other animals by making more fruits accessible and detectable, an often-overlooked consequence. While some studies of non-lethal flying fox deterrents accounted for bird damage (Raharimihaja et al., 2016; Tollington et al., 2019; Oleksy et al., 2021), others excluded it from their data (Singh, Singh & Singh, 2022). For instance, Oleksy et al. (2021) found that bird damage (by ring-necked parakeets (*Alexandrinus krameri*), red-whiskered bulbuls (*Pycnonotus jocosus*), and common mynas (*Acridotheres tristis*)) was higher on netted trees than on non-netted ones, because birds could enter through gaps in the nets. Netting orchards can also affect the orchard microclimate, reducing the risk of sunburn but potentially increasing the incidence of fungal diseases (McCaskill et al., 2016). Hence, to identify and recommend the most effective non-lethal deterrents, it is necessary to account for both flying fox damage and other sources of fruit loss to ensure robust and context-relevant management strategies.

Mauritius exemplifies an island affected by HWC in relation to a fruit-eating bat in which the Mauritian flying fox (*P. niger*), a Mascarene endemic (Cheke & Hume, 2008), is threatened with extinction (Hutson & Racey, 2013). Following exaggerated damage claims (Florens, 2015) reaching 100% alongside an increase in growers’ complaints (Government of Mauritius, 2010; Anon, 2013; Anon, 2015a; Anon, 2015b) concerning mainly lychee (*Litchi chinensis* Sonn.) and mango (*Mangifera indica* L.) orchards, the country’s biodiversity protection law was weakened in 2015 to enable government-led mass-culling of native animals. Five annual campaigns against flying foxes have been carried out between 2015 and 2020 (Florens, 2016; Florens & Vincenot, 2018; Lakhpatwala, 2019; Chelvan, 2020). The initial mass-culling campaigns had already worsened the Red List threat category of *P. niger* from Vulnerable to Endangered (Kingston et al., 2018). Netting is the only non-lethal method scientifically assessed in Mauritius, but is often incorrectly deployed by fruit growers (Oleksy et al., 2021). Other methods such as noise, light and smoke are also used (Bhanda et al., 2025), but their effectiveness remains unknown. As native food resources become scarce due to invasive alien plants (Monty, Florens & Baider, 2013; Krivek et al., 2020) and animals (Baider & Florens, 2006; Reinegger et al., 2021; Seegobin, Oleksy & Florens, 2026) and tropical fruit markets expand (Mukhametzyanov et al., 2023), flying foxes may increasingly target commercial fruit trees, intensifying HWCs (Vincenot, Florens & Kingston, 2017; Tella et al., 2020). Consequently, it is essential to compare both the viability and adoptability of non-lethal measures for protecting fruit crops.

During the fruiting seasons of lychee (October–December) and mango (December–February), between 2018 to 2024, we first evaluated the efficacy of seven non-lethal alternative methods to protect orchards in Mauritius from flying fox damage, namely sound-light system, odour-based repellents, flags, smoke and light, sprinkler system, netted structure and exclosure with parallel cords. Optimal foraging theory suggests that animals aim to maximise their net energy gain while foraging by balancing the caloric benefits of food against the costs of obtaining it (time, effort, and risk) (Pulliam, 1974; Charnov, 1976). Therefore, we hypothesised that using deterrents or mechanical barriers on commercial fruit trees would deter frugivores by increasing the costs of obtaining fruits. We predicted that frugivores would eat fewer fruits on protected trees than unprotected ones. Secondly, we investigated whether flying fox deterrent methods influenced absolute fruit loss by birds, as birds are the second most common vertebrate frugivores foraging on lychee in Mauritian orchards (Bhanda et al., 2025). Therefore, we hypothesised that reductions in flying fox damage would leave more fruits available and detectable to birds, potentially increasing bird damage and reflecting competitive release (Connell, 1961; Ruscoe et al., 2011). We also recorded other factors contributing to fruit loss in orchards.

## Materials & Methods

### Study site

Mauritius (centred around 20°20′S and 57°34′E; 1,865 km^2^; highest elevation 828 m) is a 7.8 M year-old volcanic island in the Indian Ocean (900 km east of Madagascar). It has a mean annual temperature of 22 °C and a mean annual rainfall of 800–4,000 mm (Staub, Stevens & Waylen, 2014). Mauritius is located within one of the world’s biodiversity hotspots (Myers et al., 2000) and retains only 4.4% of its native terrestrial habitat (Hammond et al., 2015) in a highly fragmented state (Florens, 2013) due to habitat destruction mainly for agriculture and urban development. Lychee and mango are the third and fourth most-produced fruits on Mauritius, playing key roles in the local market and export industry (Statistics Mauritius, 2023). The yearly production of lychee normally varies between 1,200–3,000 tonnes (Agromoris, 2023), but it can reach 15,000 tonnes (Fresh Plaza, 2016). The area under lychee cultivation covers 96 hectares, representing 8% of the total land devoted to fruit and nut production in Mauritius, while mango occupies 83 hectares, accounting for 7% (Statistics Mauritius, 2023).

### Experimental setup

**Figure 1:**
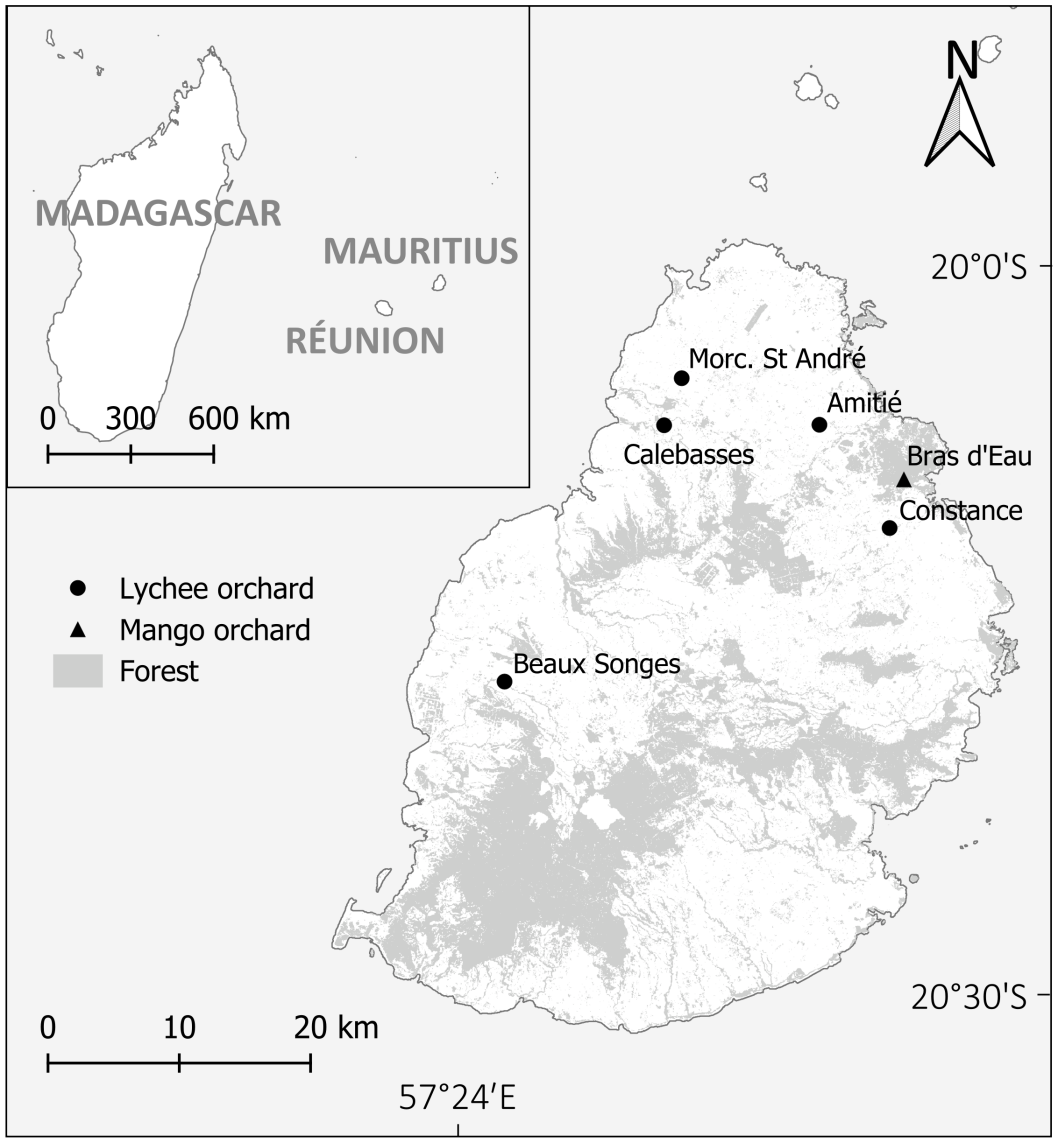
Mauritius in the Indian Ocean (insert) and location of studied orchard sites. Morc. stands for Morcellement.

**Figure 2:**
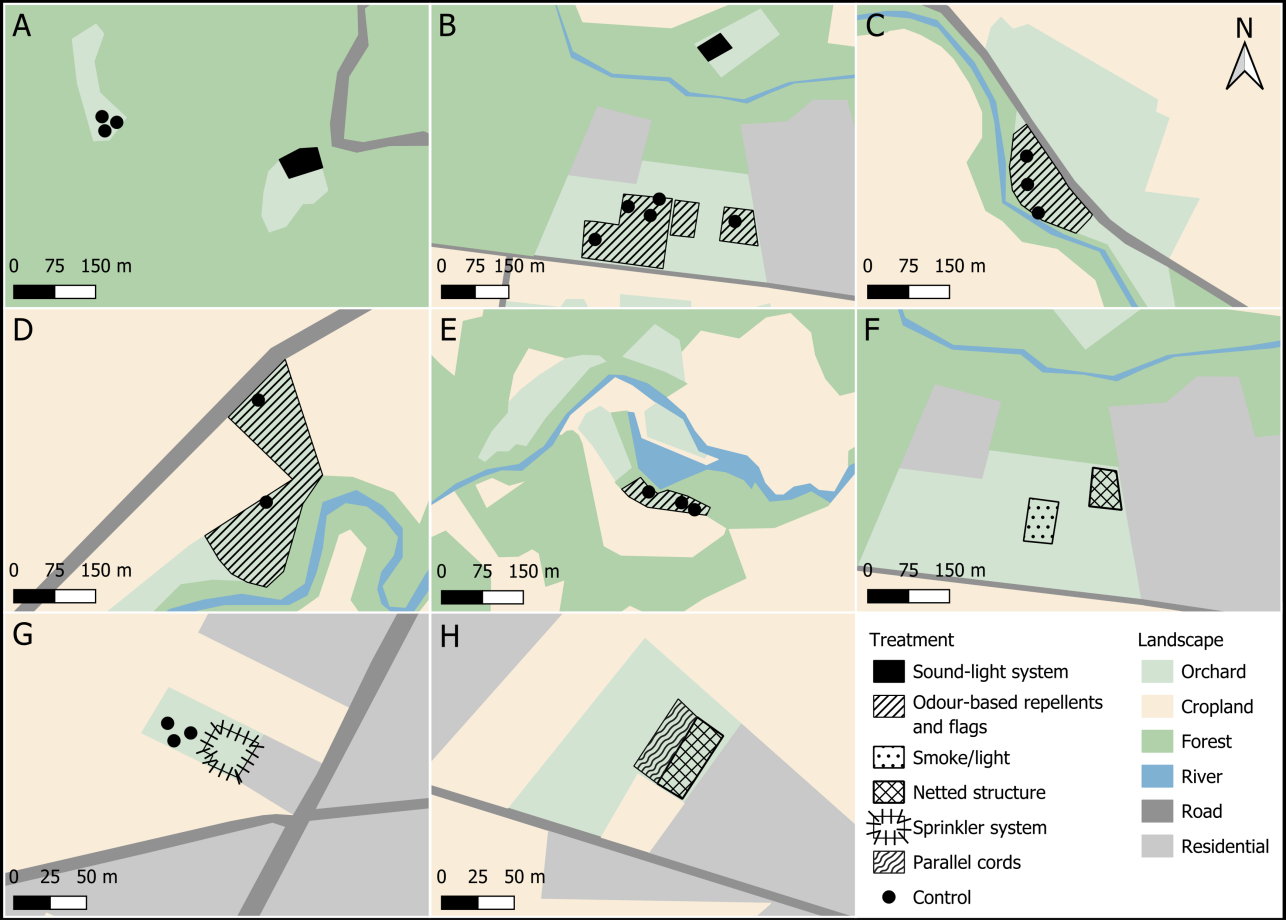
Spatial arrangement of non-lethal flying fox deterrent experimental treatments assessed in mango (panel A) and lychee (panels B–H) orchards. (A) Bras d’Eau, 2018; (B) Calebasses, 2018; (C) Beaux Songes, 2018; (D) Constance, 2018, next to road; (E) Constance, 2018, by river, one km from road; (F) Calebasses, 2021; (G) Morcellement St. André, 2024; (H) l’Amitié, 2023, 2024. Polygons show the positions of the different treatments (see key). Dots show the location of unprotected (control) trees.

With permission from the Ministry of Agro-Industry and Food Security (NP 46/3V6) and approval from the University of Mauritius Research Ethics Committee (UoMREC/2025/P90), we sampled five lychee orchards in different regions of the island over a 7-year period, including Beaux Songes (west, two hectares), Calebasses (north, nine hectares), Constance (east, three hectares), Amitié (north, 0.3 hectare) and Morcellement St André (north, 0.3 hectare) and one mango orchard at Bras d’Eau (east, two hectares) (Fig. 1). Lychee orchard owners at Beaux Songes, Calebasses and Constance were large producers, each having between 100–200 trees, whereas Amitié and Morcellement St André were small producers, each with 25–30 lychee trees. Bras d’Eau had around 70 mango trees. The study was conducted in close collaboration with orchard owners, who sometimes influenced the number and spatial arrangement of trees available for treatments and controls. All orchard trees were ∼8 m high, except ∼5 m high at Amitié. At each site, deterrent systems or protective structures were installed either at flowering or at fruit set, depending on setup requirements. Trees were randomly selected by numbering all eligible trees within the orchard and assigning treatments using a random number generator. In 2018, control trees in lychee orchards (Beaux Songes, Calebasses and Constance) were also randomly assigned, whereas at the other sites, control trees were selected based on the patches made available by orchard owners (Fig. 2).

### Sound-light system

The sound-light system was an automated waterproof system with speakers (60 W) and beacon lights (20 W) that emitted 80 different noises (*e.g.*, gunshots, firecrackers, busy traffic, dogs barking, flying fox distress calls) and flashing lights. The system was managed *via* a web-based Wi-Fi interface, allowing randomised configurations of start/stop times, speaker activity and silence durations, and light on/off periods. The system also allowed sound management through uploading and deleting audio files. Making noise (using firecrackers/gunshots, hitting empty barrels with sticks, whistling and shouting) (Bhanda et al., 2025) and illuminating tree canopies with bulbs (Oleksy et al., 2021) at night in an attempt to deter flying foxes is commonly used by orchard owners in Mauritius. Because manual noise-making requires orchard owners or hired labourers to remain in orchards overnight, the automated system offers a more practical and less labour-intensive alternative. A similar noise-based deterrent using stereo channels and speakers to randomly broadcast sounds such as motorbikes, gunshots and cries of wounded flying foxes was trialled in Australia in the late 1990s. Although initially promising, it was not adopted by the industry, which instead called for government trials that were never implemented (G.C. Richards, cited in Aziz et al., 2016).

We assessed the sound-light system using five speakers with four beacons (Appendix S1A) with a random activation period of five to seven seconds for speakers (followed by one to two seconds of silence) and one to three seconds for beacons (followed by one to three seconds with no light emission) between dusk (∼18:30) and dawn (∼05:00). The assessment was done in 2018 at a lychee orchard at Calebasses and a mango orchard at Bras d’Eau, where treatments and controls consisted of adjacent trees at one end of two larger orchards (∼300 m away). The lychee orchard was surrounded mainly by anthropogenic features, including a road, residential areas, cropland, and a small forest patch, whereas the mango orchard was bordered by a national park and a road with almost no night-time traffic. For the lychee orchard, there were 20 treatment trees and five control trees about 300 m away (Fig. 2B). The control trees used at the lychee orchard for the sound-light system were the same trees used as control for the odour-based repellents in 2018, and both control and odour-based repellent treated trees were located within the same area (Fig. 2B). For the mango orchard, data were collected on six treatment trees and three control trees about 300 m away (Fig. 2A). The trees assessed at Calebasses were occasionally exposed to active daytime deterrence, including clapping, shouting and chasing birds, whereas Bras d’Eau orchard experienced minimal human disturbance.

### Odour-based repellents and flags

The odour-based repellents consisted of four organic, water-resistant commercial deer repellents: I Must Garden^®^ (Cr1), Repels-All^®^ (Cr2), Liquid Fence^®^ (Cr3), and Deer Off^®^ (Cr4), formulated with single or combined ingredients such as mint, dried blood or putrescent egg scent. Each commercial repellent was used at the concentration recommended by the manufacturer (Appendix S1A). A homemade repellent (Hr) was also assessed, containing a blended solution of fresh strong-scented herbs (Appendix S1A). Flags were made of fabric (tetron, 1.0 × 0.6 m) attached to wooden poles 2 to 3 m high. They were then drenched in the solution, left to dry overnight and positioned just above the tree canopy using cable ties (four flags per tree). The repellent solutions were reapplied monthly. The repelling effect of flags alone without the repellent solution was also assessed, and flags with and without repellent were compared to control trees. Each group of four flags was assumed to protect only the tree on which it was placed, thereby minimising potential confounding effects between treatments and controls. Data were collected in 2018 at three lychee orchards: Beaux Songes (Fig. 2C), Calebasses (Fig. 2B) and two sites at Constance (Figs. 2D, 2E). We initially planned five replicates of each of the six treatments and of unprotected trees at each site. However, some flags were removed or trees later netted by farmers, resulting in three controls and 21 treatment trees at Beaux Songes, five controls (same control trees as the sound-light system) and 27 treatment trees at Calebasses, and five controls and 30 treatment trees at Constance.

### Smoke and light

Creating dense smoke at night by burning dry leaves and tyres (Appendix S1A) under trees is a common practice in Mauritian orchards (Bhanda et al., 2025). Since this method was not implemented by our team, identifying a site using smoke consistently and without other deterrents was challenging. In 2021 at Calebasses, we found a lychee orchard owner using smoke combined only with light, maintaining consistent smoke from dusk to dawn using paid labour and placing one 12 W bulb per tree canopy. While some orchard owners also burn dried chillies, only leaves and tyres were used in this case. Seven trees were assessed (Fig. 2F). Because smoke spread beyond the treatment area, it was not feasible to have control trees nearby; even trees farther away were unavailable because farmers were unwilling to leave them unprotected. This method was not replicated in subsequent fruiting seasons because the orchard owner switched to other deterrents and we could not find another site using this method where the owners would allow damage assessments.

### Sprinkler system

The sprinkler system was an automated device dispensing water above the tree canopy, with activation times and random parameters configurable *via* any Wi-Fi–enabled smart device with a web browser. One sprinkler per tree was used, delivering water at 2 to 4 bar over a radius of 8 to 10 m from dusk to dawn, with random activation for 60 to 90 s followed by a 90-second pause. The system was installed in a lychee orchard at Morcellement St. André in 2024 (*n* = 4 trees). Because the sprinkler’s radius matched the canopy of individual trees, only the tree with the sprinkler was protected, allowing control trees (*n* = 3) elsewhere in the orchard to be included (Fig. 2G). This method was designed in 2023 for assessment in 2023 and 2024. However, low fruit set across the island in 2023 limited year replication. The orchard at Amitié was an exception, producing enough fruit to enable assessment of the netted structure and parallel cords in 2023. This approach was similar to canopy-mounted sprinklers used in Australia in 2018 to prevent flying foxes from roosting in residential areas (Mo et al., 2020).

### Netted structure

The netted structure consisted of an exclosure covering the entire orchard, supported by poles and thick cords. White nets (mesh size 15 × 15 mm, 45 g/m^2^, with three years of UV protection) were used on the vertical sides and above the tree canopies (Appendix S1A). This method was assessed in lychee orchards at Calebasses in 2021 (*n* = 5 trees, Fig. 2F) and Amitié in 2023–2024 (*n* = 5 trees per year, Fig. 2H). Control trees could not be included because farmers were unwilling to leave any trees unprotected. The trees protected by smoke at the same orchard in 2021 were located farther away from the netted structure, so any potential effect of the smoke on the trees within the netted structure was negligible, as confirmed by site visits at night. While Oleksy et al. (2021) evaluated netting of individual trees in Mauritius, we aimed to assess the feasibility of netting an entire orchard to address orchard owners’ concerns about the labour-intensive and time-consuming nature of individual tree netting. A netted structure would provide permanent protection, requiring only occasional maintenance, compared to the seasonal installation and removal of individual nets.

### Parallel cords

The parallel cord method was a protective structure enclosing the orchard, similar to the netted structure but with the canopy covered by parallel lacing cords (black, two mm thick, UV-resistant) instead of nets. The cords were spaced 15 cm apart and installed perpendicular to the orchard’s longest side (Appendix S1A). White nets with similar specifications to those used in netted structures were installed on the vertical sides. This method was recommended for assessment by some fruit-grower members of the Human Bat Conflict Working Group of Mauritius (established in 2022), a committee of all the major stakeholders (from Government to fruit growers and conservation non-governmental organization’s (NGO’s)) concerned with mitigating or resolving the HWC. It was previously trialled by an orchard owner, who suggested it as a more durable and cost-effective alternative to netted structures. A similar approach was tested in Japan to protect orchards from crows (*Corvus* spp.), where transparent nylon lines were spaced one m apart, corresponding to the birds’ wingspan (Yoshida, Saeki & Momose, 2019). Unlike birds, flying foxes can grasp or crawl along structures using their claws, making a spacing equivalent to their wingspan unlikely to be effective. The orchard owner initially trialled the parallel cord method with a 30 cm spacing, but reported that a few flying foxes became entangled on the lines, prompting us to reduce the spacing further for the experiment. The method was assessed at Amitié lychee orchard in 2023 and 2024, with five trees monitored each year. Although control trees could not be included, data collected from the adjacent netted structure at Amitié during the same period allowed a comparison to presumed complete protection (Fig. 2H).

### Damage assessment

Panicles are many-branched inflorescences, and in lychee, they are typically 11–32 cm long, bearing approximately 7–33 fruits per panicle around 2–3 weeks after the end of flowering (Menzel & Simpson, 1992). Mango panicles are 34–40 cm long (Kavitha et al., 2022) and bear approximately 11–21 fruits per panicle at a comparable stage (Kumar et al., 2023). When fruits were still unripe and before damage started, three trained observers counted the total number of panicles per tree using hand tally counters. From each tree, a total of 20 panicles were haphazardly selected (observers visually selected panicles without applying any specific criteria, such as size, height, or fruit number, to avoid selection bias), and the number of fruits per panicle was recorded. Expected fruit yield per tree was then estimated as the product of the total number of panicles per tree (averaged across three observers) and the mean number of fruits per panicle.

Before monitoring damage, we cleared the ground beneath each tree canopy of dried leaves to facilitate fruit collection. Trees were visited regularly to determine when damage began. Once damage was observed, we collected fallen fruits under each sampled tree and classified them according to the damaging agents. Animal damage was identified based on bite or feeding marks: triangular-shaped punctures on fruit or seed for flying foxes, peck or scrape marks on fruit or seed for birds (*A. krameri*, *P. jocosus*, and *A. tristis*, identified during sampling), small incisor marks on seeds for rats (*Rattus* spp.), and incisor marks on fruit or seed for long-tailed macaques (*Macaca fascicularis*) (Bhanda et al., 2025). Other types of damage were also recorded (sunburn, fungal diseases, fruit cracking, and natural fruit fall (Appendix S1A)). Unclassified damage per tree was negligible (0.1–1%). Although a fruit could show signs of more than one type of damage, each fallen fruit was attributed to a single cause of damage, and no fruit was counted more than once. For instance, sunburn-affected fruits that remained on the tree (and could still be harvested) but were later consumed and dropped by flying foxes were attributed to flying fox feeding, whereas cracked fruits subsequently fed on by birds were classified as fruit cracking (characteristic cracking on the skin remained visible), as these fruits would not be harvested regardless of secondary damage. Although macaques and rats could carry fruits away from the tree (Bindra, 1948; Corlett & Lucas, 1990), the main species of interest (flying foxes and birds) are known to feed directly on fruits in the canopy (Oleksy et al., 2021). Damage assessments continued throughout the fruiting season (November–December for lychee and December–January for mango) for four consecutive weeks, until harvest. Effectively protected trees were harvested during the fifth week. The percentage of fruit damage or removal attributable to each source, including animal agents and harvest, was calculated using Eq. (1).

Equation (1). Percentage of fruits affected by source *x* on tree *i* (*PD*_*i*,*x*_), calculated as the ratio of the total number of fruits affected by source *x* on tree *i* (*N*_*i*,*x*_) to the expected fruit yield of tree *i* (${\hat {Y}}_{i}$), multiplied by 100.

(1) \begin{eqnarray*}P{D}_{i,x}= \frac{{N}_{i,x}}{{\hat {Y}}_{i}} \times 100.\end{eqnarray*}

### Statistical analyses

All statistical analyses were conducted in R (version 4.4.2) (R Core Team, 2025). We tested the effects of different deterrent methods on the proportions of fruits eaten by flying foxes and birds per tree with Generalized Linear Models (GLMs), weighting the data by fruit yield per tree to account for the number of expected fruits contributing to each proportion (using package glmmTMB—Brooks et al., 2023). We tested the effect of each deterrent method with a separate GLM, comparing treatment and control trees, with each tree treated as an individual data point because data for different deterrents were collected at different sites and years. In each GLM, we included a two-way interaction between the deterrent methods and tree species (sound-light system), site (odour-based repellents and flags) or year (netted structure and parallel cords). We initially fitted GLMs with a binomial error distribution because our response variables are proportions derived from counts (Douma & Weedon, 2019). Model assumptions and potential misspecification were assessed using simulated standardized quantile–quantile (QQ) and residuals plots generated with the package DHARMa (Hartig, 2024). Residual diagnostic plots indicated overdispersion. Therefore, we refitted GLMs with a beta-binomial error distribution as recommended by Douma & Weedon (2019), successfully accommodating the overdispersion. Despite the low n/k ratio of some of our GLMs (4–7), they still provided reasonable estimates with small standard errors.

We then tested each GLM against a null model with a likelihood ratio test, because this provides a robust measure of how likely significant effects arise by sampling variation alone (Forstmeier & Schielzeth, 2011). We reported the model estimates and carried out *post hoc* contrast tests for the pairwise comparisons between deterrent methods for each site or year, correcting for multiple comparisons using the Tukey method (package emmeans—Lenth et al., 2023). We evaluated statistical significance of model estimates using 95% confidence intervals (CIs) (Nakagawa & Cuthill, 2007). Due to low sample sizes, results for the smoke/light method and sprinkler system were reported descriptively. Partial Spearman rank correlations were performed to assess the relationship between the proportion of fruit damaged by flying foxes and by birds while controlling for fruit yield per tree, with 95% CIs (using package ppcor Kim, 2015). Analyses were conducted separately for each site within a deterrent method to account for site-level variation in damage patterns. To avoid duplicate comparisons, control trees that were shared between the sound-light system and odour-based repellents and flags at Calebasses in 2018 were included only once in the correlation analysis.

## Results

### Sound-light system

**Figure 3:**
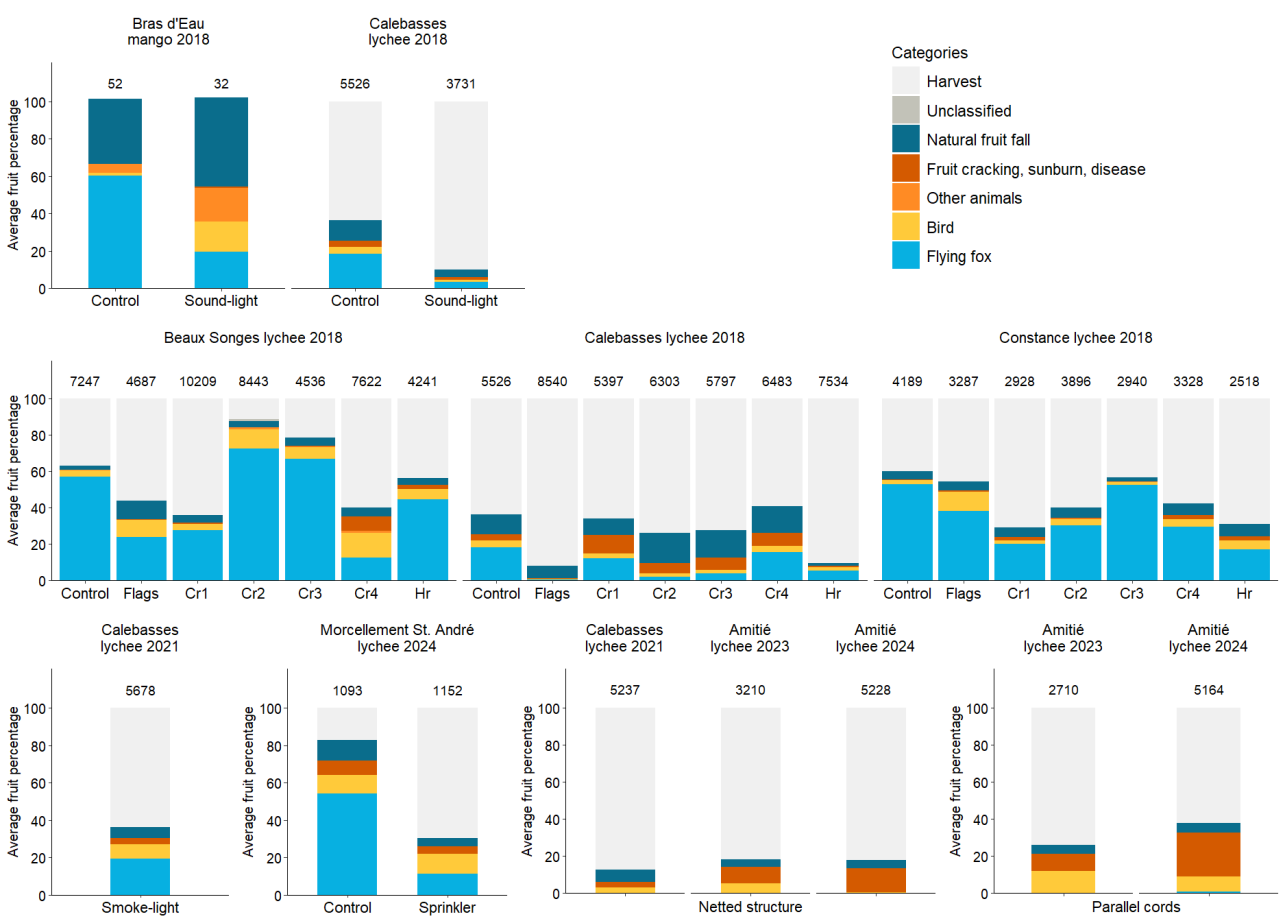
Percentage of fruits damaged and harvested under different flying fox deterrent experimental treatments. Stacked bars represent the average percentage of fruits per tree harvested and lost to different factors across treatments, sites, and years (see key). The “Other animals” category includes losses caused by rats, macaques, and fruit flies. Numbers above bars indicate the average number of fruits per tree. All mangoes at Bras d’Eau were damaged before they could be harvested.

Lychee trees at Calebasses bore 5,526 ± 2,179 fruits (mean ± SD) on unprotected trees and 3,731 ± 2,297 fruits on trees protected by the sound-light system. An average of 2,094 ± 1,568 (36%) lychees were damaged on unprotected trees, and 368 ± 414 (10%) on protected trees (Fig. 3). Flying fox damage at the lychee orchard was significantly lower on trees protected with the sound-light system (4 ± 6%) compared to control trees (18 ± 15%) (Table 1). Calebasses was the first orchard in Mauritius where this deterrent system was deployed. A few nights after installation, the system stopped working for one night due to technical issues, which were resolved before the following night. However, during both the first and second weeks of assessment, flying fox damage averaged 0%, increasing to 1 ± 1% in the third week and 2 ± 5% in the fourth week, indicating that the one-night interruption was unlikely to have influenced the results. Bird damage was also significantly lower on protected trees (1 ± 1%) compared to control trees (4 ± 2%) (Table 1). At the lychee orchard, flying fox and bird damage were positively correlated (Table 2). Rat damage was negligible (<1%). Lychees damaged by sunburn, disease and fruit cracking accounted for 2 ± 3% and natural fruit fall was 5 ± 5%.

Mango trees at Bras d’Eau bore 52 ± 30 fruits on unprotected trees and 32 ± 8 fruits on protected trees. Of these, 53 ±  31 (101%, indicating more fruits were collected from the ground than originally estimated on the tree) mangoes were damaged on unprotected trees, and 33 ± 8 (102%) were damaged on protected trees (Fig. 3). The percentage of mangoes damaged by flying foxes at Bras d’Eau was significantly lower on protected trees (20 ± 9%) compared to control trees (60 ± 12%) (Table 1). For birds, damage to mangoes was significantly higher on protected trees (16 ± 8%) compared to control trees (1 ± 3%) (Table 1). There was a negative correlation between flying fox and bird damage at the mango orchard (Table 2). Macaque damage was 18 ± 10% on protected mango trees and 5 ± 4% on control trees. Mangoes damaged by disease and fruit cracking were recorded only on protected trees (1 ± 2%). Natural fruit fall was on average 59 ± 18%.

### Odour-based repellents and flags

Lychee trees produced 7,247 ± 3,227 fruits at Beaux Songes, 5,526 ± 2,179 fruits at Calebasses and 4,189 ± 2,418 fruits at Constance for control trees, while trees protected with odour-based repellents and flags produced on average 6,423 ±3,761, 6,686 ± 2,432 and 3,149 ± 1,800 fruits, respectively. Fruit loss from non-protected trees amounted to 3,599 ± 3,052 (63%), 2,094 ± 1,568 (36%), and 2,428 ± 3,206 (60%) fruits at Beaux Songes, Calebasses and Constance, respectively, and trees protected with odour-based repellents and flags was 3,257 ± 3,133 (55%), 1,244 ±1,469 (25%), and 1,434 ± 2,159 (42%), respectively (Fig. 3). Because no consistent pattern emerged among the five repellents, we reported their average values only. Non-protected trees at Beaux Songes, Calebasses and Constance sustained flying fox damage of 57 ±47%, 18 ± 15%, and 53 ± 49%, respectively, whereas trees protected with the odour-based repellents experienced an average damage of 43 ± 38% at Beaux Songes, 8 ± 14% at Calebasses and 30 ± 38% at Constance. Flying fox damage for trees protected with flags only was 24 ± 24% at Beaux Songes, 1 ± 1% at Calebasses and 38 ± 37% at Constance. At Constance, some trees were situated by a main road, while others were one km away, along a river (Fig. 2). For trees located near the river, flying foxes damaged on average 88 ± 9% (*n* = 3) of fruits on control trees, 82 ± 11% (*n* = 8) on trees protected with odour-based repellents, and 70 ± 20% (*n* = 2) on trees protected with flags only. Control trees close to the main road sustained no flying fox damage (*n* = 2), whereas trees protected with odour-based repellents and flags only sustained 17 ± 30% (*n* = 3) and 5 ± 10% (*n* = 17) flying fox damage, respectively.

**Table 1:** Effects of deterrent experimental treatments on the proportion of fruit damaged by flying foxes and birds. The results of Generalized Linear Models with beta-binomial error distribution explaining the effects of different deterrents (sound-light system, odour-based repellents, flags, netted structure and parallel cords) on the proportion of Fruits eaten by flying foxes and birds. Mango orchards are denoted by (m) and lychee orchards by (l). Sample sizes (*n*) represent the number of trees surveyed at each site. Regression coefficients, standard errors (SEs) and 95% Confidence Intervals (CIs) are provided for pairwise comparisons between the control or netted structure and deterrent methods at each site and year. Regression coefficients are provided in the following format: Coefficient ± SE (CI_low_, CI_up_). Results are given on the response scale (proportions). Effects for which the 95% or 99% CIs do not overlap zero are written in bold.

Deterrent method Site, year Deterrent comparison		Response variable			
		Proportion of flying fox eaten fruits		Proportion of bird eaten fruits	
	*n*	Coefficient ± SE (CI_low_, CI_up_)	*p*-value	Coefficient ± SE (CI_low_, CI_up_)	*p*-value
**Sound-light system**					
Bras d’Eau (m) 2018	9				
Control *vs.* sound-light		**0.40 ± 0.10 (0.20, 0.60)**	**<0.01**	**−0. 15 ± 0.03 (−0.21, −0.08)**	**<0.01**
Calebasses (l) 2018 Control *vs.* sound-light	25	**0.12 ± 0.04 (0.04, 0.20)**	**<0.01**	**0.03 ± 0.01 (0.01, 0.04)**	**<0.01**
**Odour-based repellents and flags**					
Beaux Songes (l) 2018	24				
Control *vs.* Cr_1_		0.20 ± 0.23 (−0.46, 0.87)	0.97	0.02 ± 0.03 (−0.07, 0.12)	0.99
Control *vs.* Cr_2_		−0.13 ± 0.28 (−0.94, 0.69)	1.00	−0.06 ± 0.06 (−0.25, 0.13)	0.96
Control *vs.* Cr_3_		0.11 ± 0.25 (−0.62, 0.84)	1.00	−0.05 ± 0.05 (−0.18, 0.09)	0.96
Control *vs.* Cr_4_		0.26 ± 0.22 (−0.40, 0.92)	0.91	0.00 ± 0.04 (−0.11, 0.11)	1.00
Control *vs.* Hr		0.07 ± 0.26 (−0.68, 0.83)	1.00	−0.03 ± 0.05 (−0.16, 0.11)	1.00
Control *vs.* flags only		0.20 ± 0.22 (−0.44, 0.84)	0.97	0.00 ± 0.04 (−0.11, 0.11)	1.00
Calebasses (l) 2018	32				
Control *vs.* Cr_1_		0.15 ± 0.15 (−0.30, 0.60)	0.95	0.04 ± 0.03 (−0.04, 0.13)	0.79
Control *vs.* Cr_2_		0.12 ± 0.15 (−0.31, 0.56)	0.98	0.02 ± 0.03 (−0.07, 0.11)	0.99
Control *vs.* Cr_3_		0.12 ± 0.15 (−0.33, 0.58)	0.98	0.03 ± 0.03 (−0.06, 0.12)	0.96
Control *vs.* Cr_4_		0.15 ± 0.15 (−0.29, 0.58)	0.96	0.03 ± 0.03 (−0.06, 0.12)	0.94
Control *vs.* Hr		0.14 ± 0.14 (−0.29, 0.56)	0.96	0.04 ± 0.03 (−0.05, 0.12)	0.82
Control *vs.* flags only		0.22 ± 0.14 (−0.18, 0.62)	0.67	0.05 ± 0.03 (−0.03, 0.13)	0.54
Constance (l) 2018	35				
Control *vs.* Cr_1_		−0.03 ± 0.15 (−0.46, 0.40)	1.00	−0.02 ± 0.02 (−0.07, 0.04)	0.98
Control *vs.* Cr_2_		0.04 ± 0.14 (−0.36, 0.44)	1.00	−0.01 ± 0.02 (−0.06, 0.04)	1.00
Control *vs.* Cr_3_		0.00 ± 0.16 (−0.46, 0.46)	0.99	0.00 ± 0.02 (−0.05, 0.04)	1.00
Control *vs.* Cr_4_		−0.12 ± 0.17 (−0.61, 0.36)	1.00	−0.03 ± 0.02 (−0.10, 0.04)	0.78
Control *vs.* Hr		0.02 ± 0.14 (−0.40, 0.44)	1.00	−0.02 ± 0.02 (−0.07, 0.04)	0.98
Control *vs.* flags only		0.03 ± 0.15 (−0.40, 0.46)	1.00	−0.01 ± 0.02 (−0.08, 0.05)	0.99
**Netted structure and parallel cords**					
Amitié (l) 2023					
Netted structure *vs.* parallel cords	10	0.00 ± 0.00 (0.00, −0.01)	0.10	**−0. 07 ± 0.03 (−0.01, −0.13)**	**0.02**
Amitié (l) 2024					
Netted structure *vs.* parallel cords	10	−0.01 ± 0.00 (0.00, −0.01)	0.05	**−0. 08 ± 0.02 (−0.04, −0.13)**	**<0.01**

**Table 2:** Relationship between flying fox and bird damage under the different flying fox deterrent methods. Partial Spearman rank correlation between the proportion of flying fox and bird damage, controlling for estimated fruit yield per tree, across deterrent methods and sites. Mango orchards are denoted by (m) and lychee orchards by (l). The number of trees (*n*), correlation coefficient (*ρ*), and *p*-values are reported. Statistically significant relationships are indicated in bold. The netted structure was not included because it was designed to exclude both flying foxes and birds.

Deterrent method Site	*n*	Correlation coefficient (*ρ*)	*p*-value
Sound-light system			
Bras d’Eau (m)	9	**<0.01**
Calebasses (l)	20	**0.64**	**<0.01**
Odour-based repellents and flags			
Beaux Songes (l)	24	0.38	0.08
Calebasses (l)	32	**0.83**	**<0.01**
Constance (l)	35	**0.76**	**<0.01**
Smoke and light			
Calebasses (l)	7	−0.27	0.61
Sprinkler system			
Morcellement St. André (l)	7	0.45	0.37
Parallel cords			
Amitié (l)	10	0.12	0.77

Bird damage was 4 ± 3%, 4 ± 2% and 2 ± 3% on control trees; 7 ± 9%, 2 ± 3% and 3 ± 5% on trees protected with odour-based repellents; and 9 ± 8%, <1% and 10 ± 15% on trees protected with flags only at Beaux Songes, Calebasses and Constance, respectively. There was no significant difference in flying fox or bird damage between control trees and trees protected with odour-based repellents, or control trees and trees protected with flags only (Table 1). There was strong evidence of a positive correlation between flying fox and bird damage at Calebasses and Constance (Table 2). Macaque damage was recorded at Beaux Songes only (<1%), and rat damage was negligible (<1%) at all three sites. Fruit loss due to sunburn, disease and fruit cracking was on average 2 ± 5% at Beaux Songes, 5 ± 10% at Calebasses and 1 ± 2% at Constance and natural fruit fall was 5 ± 6%, 11 ± 13% and 5 ± 7%, respectively.

### Smoke and light

Lychee trees at Calebasses produced 5,678 ± 3,193 fruits on trees protected with the combination of smoke and light method. On average, 2,078 ± 1,197 (36%) fruits were damaged on protected trees (Fig. 3). Since there were no control trees for comparison, no statistical analysis was performed. Flying fox and bird damage were 19 ±  9% and 8 ± 6%, respectively, on protected trees. There was a negative correlation between flying fox and bird damage, but this relationship was not statistically significant (Table 2). Rat damage was negligible (<1%). Fruit loss due to sunburn, disease and fruit cracking was 3 ± 3% and natural fruit fall accounted for 6 ± 3% of fruits on protected trees.

### Sprinkler system

Lychee trees at Morcellement St. André bore 1,198 ± 99 fruits on unprotected trees and 1,152 ± 797 fruits on trees protected with sprinklers. Of these, 1,010 ± 405 (83%) fruits were damaged on unprotected trees and 379 ± 272 (30%) on protected trees (Fig. 3). Control trees sustained 54 ± 10% flying fox damage compared to 11 ± 10% for protected trees. Bird damage was 10 ±  7% on control trees and 11 ± 9% on protected trees. However, due to the small sample size, statistical comparisons between treatment and control trees were not performed. There was a positive correlation between flying fox and bird damage, but this relationship was not statistically significant (Table 2). Rat and fruit fly damage was negligible (<1%) at Morcellement St. André. Sunburn, disease and fruit cracking accounted for 8 ± 6% loss on unprotected trees and 4 ± 3% on protected trees. Natural fruit fall was 11 ± 10% on unprotected trees and 4 ± 4% on protected trees.

### Netted structure

Lychee trees protected by netting produced 5,237 ± 2,185 fruits at Calebasses in 2021, 3,210 ± 1,833 fruits at Amitié in 2023, and 5,228 ± 1,483 fruits at Amitié in 2024. Of these, damaged fruits amounted to 738 ± 713 (13%), 338 ± 192 (18%) and 731 ± 837 (18%) in 2021, 2023 and 2024, respectively (Fig. 3). Flying fox damage was negligible at Calebasses in 2021 (0.1%) and absent at Amitié in both 2023 and 2024. Bird damage was 3 ± 3% in 2021, 5 ± 7% in 2023, and 1 ± 1% in 2024 (Fig. 3), due to holes in the nets. Rat damage was negligible and was recorded only at Calebasses. The combination of sunburn, disease and fruit cracking affected 3 ± 3% of fruits in 2021 (Calebasses), 9 ± 11% in 2023 (Amitié) and 12 ± 16% in 2024 (Amitié). Natural fruit fall was 7 ± 3%, 4 ± 5% and 4 ± 4% in 2021, 2023 and 2024, respectively.

### Parallel cords

Lychee trees protected by parallel cords at Amitié produced 2,710 ± 1,515 fruits in 2023 and 5,164 ± 1,083 fruits in 2024. On average, fruit loss was 540 ± 78 (26%) in 2023 and 2,023 ± 1,118 (38%) in 2024 (Fig. 3). Flying fox damage was negligible (<1%) in 2023 and 1 ± 1% in 2024. Strong winds occasionally moved and intertwined the cords, creating wider gaps that allowed flying foxes access and resulted in recorded damage. The cords were subsequently straightened on each occasion. Bird damage was 12 ± 11% in 2023 and 8 ± 1% in 2024. Bird damage was significantly higher on trees protected with parallel cords than on those protected with nets at Amitié, with moderate evidence of this effect in 2023 and strong evidence in 2024 (Table 1). The positive correlation between flying fox and bird damage was not statistically significant (Table 2). Rat and fruit fly damage under parallel cords was negligible (<1%) in both years. Sunburn, disease and fruit cracking resulted in 9 ± 5% damage in 2023 and 23 ± 18% in 2024. Natural fruit fall was 5 ± 3% in 2023 and 5 ± 2% in 2024. In 2024, fruit cracking increased from <1% to a maximum of 42% (the highest recorded per tree) under parallel cords and to a maximum of 39% under the netted structure following heavy rainfall associated with a cyclone. Fruit damaged by diseases remained <1% before and after the cyclone.

## Discussion

### Flying fox and bird damage

Protecting fruit trees from damage has long been central to mitigating HWC in Mauritius. However, protection has become increasingly critical over the past decade as damage to unprotected trees has intensified. In 2015, Oleksy, Racey & Jones reported flying fox damage of 53% at Beaux Songes and 9% at Calebasses. By 2018, damage at these sites had increased to 57% and 18%, respectively (our study), and by 2022, it reached 72% at Beaux Songes and 85% at Calebasses (Bhanda et al., 2025). Bird damage at the same sites was 3–8% in 2015 and 2018 and increased to 10% at Beaux Songes and 23% at Calebasses in 2022. This escalation is likely in part linked to the increasing adoption of netting, which may concentrate frugivory on the remaining unprotected trees and in part linked to a displacement of flying foxes to forage more often outside native forests because of habitat degradation by alien species. Invasive alien plants reduce fruit production of native trees (Krivek et al., 2020; Seegobin, Oleksy & Florens, 2024), and the progressing invasion in native habitats (Florens et al., 2016) is resulting in an excess mortality of larger native forest trees (≥10 cm in trunk diameter) at a rate that extrapolates to ∼100,000 trees per year over the island’s remaining native forests (Florens et al., 2017b). Flying fox damage on unprotected trees during our study exceeded 50% at most sites, except at Calebasses (<20%) and for trees located next to the main road at Constance (0%). Low flying fox damage at Calebasses (9%) was also reported in 2015, suggesting site-specific effects. Calebasses comprises numerous small orchards owned by different growers, resulting in higher nocturnal human activity that could have influenced flying fox foraging behaviour. At Constance (by the road), high nocturnal human activity was also likely, as both protected and unprotected trees located near the river sustained 75% more flying fox damage than those adjacent to the main road. Although yield was higher at Beaux Songes and Calebasses than at Constance, flying fox damage was greater at Beaux Songes and Constance by the river, likely because these orchards were farther from residential areas and the trees assessed were closer to rivers. This pattern may reflect flying fox use of rivers as navigational features, as reported in Australia (Palmer & Woinarski, 1999) and Thailand (Thanapongtharm et al., 2015), as well as their tendency to roost near prominent watercourses (Hall & Richards, 2000), placing orchard areas closer to waterways at greater risk of flying fox frugivory.

In this study we tested seven potential non-lethal methods for mitigating damage to lychee and mango fruits from vertebrate frugivores, especially flying foxes and birds. Despite limited sample sizes within locations and limited replication among locations and years, we suggest that some clear patterns have been revealed. In the following paragraphs, we discuss each of these methods in order of increasing average damage.

The lowest level of fruit damage was, as expected, in the trees within netted exclosures. Average damage from flying foxes in three location-years was essentially zero. Bird damage also occurred due to damage to the nets, but remained low (3%, based on the mean damage across site-years). Because control trees were not available for this method, the expected level of flying fox damage was inferred from untreated control trees at other sites. We used the average of mean flying fox damage across all control sites, excluding Calebasses and Constance by the road, as these sites may not have been representative of actual flying fox damage. Expected flying fox damage was 66%, indicating a reduction of more than 60% under netted exclosures. In contrast, expected bird damage (average of mean across all control sites) was 5%, suggesting little net effect on bird damage. During the 2015 lychee season, Oleksy et al. (2021) evaluated the effectiveness of netting individual trees and reported a decrease in flying fox damage to <1% at Calebasses and 13% at Beaux Songes. In orchards in the United States, it has been reported that repeated seasonal deployment and removal can cause more damage to nets, suggesting that permanent netted structures represent a more durable and easily maintained alternative (Taber, 2002).

Exclusion netting requires a relatively high initial investment (Fig. S1), and during the installation of the netted structure, we observed that the newly purchased nets could tear easily, requiring additional time to patch the holes. To reduce handling-related damage and improve efficiency, higher quality nets should be favoured, and future net installations could be mechanised, for instance by using drones to position nets over trees, a strategy that emerged during the 2024 fruiting season in Mauritius. Another alternative could be to use a more durable material, such as chicken wire, for the protective structure (Fig. S2). Improper net installation, including nets placed in contact with the canopy, would allow flying foxes to enter through gaps in the nets or to access fruits from outside by using the nets as support, which can lead to new holes as flying foxes chew fruits through the nets. Hence, nets should be installed without openings and with a gap of at least 50 cm (MWF, 2019) maintained between the net and the tree canopy (Fig. S2). The use of white nets instead of black ones (Fig. S2) may also help reduce flying fox entanglement (Charerntantanakul, Shibata & Vincenot, 2023), as lighter colours provide greater visual contrast under low-light conditions and are therefore likely more detectable to flying foxes at night (Müller, Goodman & Peichl, 2007). Nonetheless, black nets are still sold and used in Mauritius. At Calebasses, some 25 flying foxes were trapped inside a single poorly (black) netted tree after one night (G. Bhanda, pers. obs., 2020). This situation is common in orchards, often leading to the illegal capture and killing of trapped or entangled flying foxes.

The use of parallel cords resulted in negligible flying fox damage (<1%) in both years, whereas bird damage was on average 10%. Since this treatment had no control trees, expected damage values, calculated in the same way as for netting, were used for comparison. Similar to netting, parallel cords reduced flying fox damage by over 60%, relative to expected damage. However, bird damage was 5% higher than in control trees. These results indicate that parallel cords spaced 15 cm apart was effective as a mechanical barrier to flying foxes, but did not reduce bird damage. Trees protected with the parallel cords sustained 8% more bird damage compared to those protected by nets, indicating that the latter provide better protection against birds. To prevent sagging and gaps by intertwining cords, the parallel cord method could be improved by substituting the material with stainless steel wires. Although the investment cost of parallel cords was slightly lower than that of a fully netted structure (Fig. S1), its installation was time-consuming, labour-intensive, hard to set up, and only practical for orchards with tree heights not exceeding five meters.

The next lowest damage occurred with the sound-light system that we developed. Flying fox damage averaged only 4% and bird damage only 1%. The sound-light system effectively reduced flying fox damage by 14% relative to control trees at that site. However, because control trees at Calebasses experienced exceptionally low flying fox damage and were also used in clustered trials of odour-based repellents, the system may achieve a reduction of up to 62% when compared with the overall expected flying fox damage. Higher flying fox damage was associated with higher bird damage. This pattern may result from active deterrence measures targeting birds in areas where protected trees were located. Although studies on the effect of noise on foraging flying foxes seemed limited, anthropogenic disturbances like traffic noise are known to decrease the activity and foraging efficiency of insectivorous bats (Luo, Siemers & Koselj, 2015; Buxton et al., 2020). The decrease in foraging efficiency was due to noise avoidance, a mechanism that could also negatively affect species that do not depend on sound to forage (Luo, Siemers & Koselj, 2015). Flying foxes in Nepal avoided anthropogenic noise by roosting at elevated heights on trees (Hyongaju et al., 2024). Artificial lights were found to deter flying foxes in lychee orchards in India, where 20-watt bulbs (similar wattage to the system used in this study) decreased damage from 6% to 3% (Singh, Singh & Singh, 2022).

We also tested the sound-light system in a mango orchard, where we found a 40% reduction in flying fox damage and 15% increase in bird damage. The smaller difference in flying fox damage observed in the lychee orchard compared to the mango orchard may be associated to differences in attractiveness of flying foxes to the different fruit tree species or to variations in the surrounding landscape. Additionally, in 2022, another mango orchard in the north implemented the sound-light system. Due to time constraints, we could not assess its effectiveness, but the orchard owner indicated that flying fox damage decreased after its installation. Lower flying fox damage was also associated with higher bird damage as the nocturnal system had no effect on birds. This supported findings from Bhanda et al. (2025) that birds were the second most common vertebrate frugivores foraging on commercial fruits. Reduced flying fox damage at night may increase fruit availability for birds during the day, or flying foxes may remove most fruits at an earlier ripeness stage, leaving fewer fruits for birds. One constraint on the application of this system is the need for an electrical supply in the orchard. Allocating the control and treatment trees in clusters approximately 300 m apart was necessary to prevent any effect of the system on unprotected trees. Since noises can disturb people at night, this system would be more suitable for orchards located at an appropriate distance from residential areas.

The sprinkler system, another method we developed, also resulted in relatively low damage, with both flying fox and bird damage averaging 11%. Because control trees were limited, expected damage levels calculated across sites were used for comparison. This resulted in a 55% reduction in flying fox damage but a 6% increase in bird damage, likely because the nocturnal system had no effect on birds. While this study was the first to assess the efficacy of sprinklers as a flying fox deterrent in orchards, canopy-mounted sprinkler trials for flying fox roost dispersal in Australia showed that flying foxes avoided the sprinkler’s target range (Mo et al., 2020). Although site-specific, this method shows potential for protecting individual trees, such as in backyard gardens, and is relatively quick and easy to install. However, replication would be valuable to confirm its broader effectiveness. This system relies on both electricity and water supply.

The combination of smoke and light, a method used by orchard owners, resulted in a flying fox damage of 19% and a bird damage of 8%. As control trees were unavailable, comparisons with expected damage on unprotected trees suggest a 36% reduction in flying fox damage but a 3% increase in bird damage likely because the nocturnal deterrence did not affect birds. In Australia, orchard owners reported that flying foxes avoided smoke (Bicknell, 2002), but the efficacy of using smoke was not systematically assessed in orchards. Although primarily studied in the context of wildfires, smoke exposure can trigger arousal from torpor in bats (*e.g.*, *Nyctophilus gouldi*: Doty et al., 2018), leading to roost abandonment. The effect of fire or smoke on flying foxes, however, remains understudied (Loeb & Blakey, 2021). In lychee orchards in India, 12-watt bulbs (similar wattage to those from the smoke-light deterrent) resulted in a relatively low flying fox damage (Singh, Singh & Singh, 2022). However, continuous exposure to smoke from the burning of leaves and tyres would negatively affect human health and the environment (Lewtas, 2007; Jimoda et al., 2018) and pose increased fire hazards. Burning of tyres in particular, is extremely polluting and should be strongly discouraged if not strictly banned.

Odour-based repellents resulted in flying fox damage of <10% at Calebasses and <45% at Beaux Songes and Constance, corresponding to a 10–23% reduction compared with control trees. Trees protected with flags experienced only 1% damage at Calebasses and <40% at the other two orchards, representing a 15–33% reduction relative to control trees. Bird damage averaged <5% for control trees and trees protected with odour-based repellents, and ≤10% for trees protected with flags only. Despite the decrease in flying fox damage with odour-based repellents and flags, these differences were not statistically significant. Higher flying fox damage was associated with higher bird damage, suggesting that trees not effectively protected from flying foxes may also sustain higher bird damage. One of the commercial repellents tested in Mauritius contained an active ingredient similar to the one used in Madagascar, but the application method differed. In Madagascar, the repellent was sprayed directly on fruits (Raharimihaja et al., 2016), as the product is marketed for use in orchards. In contrast, in our study, repellents were applied to flags rather than directly to fruits since not all brands recommended direct application to edible plant parts. This difference in application method may have reduced overall efficacy. Furthermore, these organic repellents are susceptible to photodegradation, with sunlight accelerating the breakdown of compounds such as mint or thyme oil (Cudlik & Buchbauer, 2020), potentially diminishing their effectiveness. Hence, future studies could focus on deterrents that can be sprayed directly on fruits, with more frequent re-application, and a larger sample size, avoiding spatial clustering of control and the different treatment trees.

### Other sources of fruit loss

Macaque damage was observed mainly in the mango orchard, whereas damage in lychee orchards was negligible. Protected mango trees sustained 13% more macaque damage than unprotected ones, suggesting that flying foxes may remove fruits that would otherwise be available to macaques. More fruits were found under some mango trees than were estimated to initially exist, a paradox apparent only at Bras d’Eau, where macaque activity was high, suggesting that macaques might have carried fruits from neighbouring trees while moving between canopies, as they often do in their native range (Tsuji & Su, 2018). The high connectivity of that orchard to forested areas, combined with minimal human disturbance, may have further promoted macaque foraging. In vegetable plantations in Mauritius, macaques foraged mainly during periods of low human presence, because they were actively chased or deterred by farmers (Vallet Adams, 2025)—a response also commonly adopted by fruit growers.

Natural fruit drop was highest in the mango orchard (>50%) compared to the lychee orchards (≤11%), exceeding the 26% loss reported by Oleksy et al. (2021) for mango trees taller than six meters (similar to those in our study). Fruit drop in lychee orchards was consistent with the findings of Oleksy et al. (2021). Low yield due to severe fruit drop is a widespread problem in mango and lychee-growing countries, with fruit loss reaching 76–100% in mango (Singh, Malik & Davenport, 2004) and <22–80% in lychee (Drinnan, 2014). Fruit cracking reached 42% at one lychee orchard following a cyclone passing 80 km offshore the island in November 2024 (European Commission ERCC, 2024). In this region, the highest 24-hour rainfall was 0–10 mm in October (Mauritius Meteorological Services, 2024a) and 120–130 mm in November–the second warmest November (maximum: 33.6 °C) since 1960 (Mauritius Meteorological Services, 2024b). Similar to our findings, fruit cracking reached 43% in India (Lal, Kumar & Pandey, 2023).

## Conclusion

Our study provided an evidence-based assessment of various non-lethal flying fox deterrent methods commonly used in orchards in a country with a recent history of worsening human-wildlife conflict over the native and endangered flying fox including commercial fruits in its diet. Netted structures afforded the most reliable crop protection, and future policies could support orchard owners in installing permanent protective structures, including exploring mechanised installation methods, which would reduce damage to nets and decrease the need for frequent replacements. Given their unrivalled effectiveness, higher quality, more durable white nets should also be encouraged. The sound-light and sprinkler systems appeared promising. Future studies with increased replication across orchards and years, particularly when multiple deterrents are strategically combined and seasonally alternated to reduce habituation, will help refine recommendations for long-term application. Smoke from burning tyres is not recommended due to health hazards and fire risk. Reductions in flying fox damage at some sites were linked to higher bird or macaque damage, emphasizing the need to consider all sources of fruit loss in such studies. Our results contrast with past lethal approaches, which failed to benefit growers while severely impacting the endangered Mauritian flying fox, a species crucial for native seed dispersal and forest regeneration. By combining evidence-based approaches with durable protective measures, this study provides actionable guidance for policy makers and farmers and contributes to conserving endangered species and mitigating human-wildlife conflicts, supporting the 2030 Global Biodiversity Framework.

##  Supplemental Information

10.7717/peerj.20859/supp-1 Supplemental Information 1 Supplementary methods information Additional details from the Methods section, including an overview of the sound-light system setup, the concentrations of odour-based repellents used, a figure showing the different non-lethal flying fox deterrent methods assessed, and a figure illustrating other types of fruit damage.

10.7717/peerj.20859/supp-2 Supplemental Information 2 Investment costs of flying fox deterrents over two fruiting seasons Investment cost of different flying fox deterrent methods tested during the study, shown for the first and second fruiting seasons (year 1 and year 2). The 100% stacked bar chart displays the proportionate cost of each deterrent type per year. Values on the chart represent estimated costs in USD (based on the exchange rate as of 29 April 2025) for 20 trees. The 100% stacked bar chart displays the proportionate cost of each deterrent type per year. Estimates include materials, equipment, and electricity and water supply, where applicable. Labour and software development costs are not included.

10.7717/peerj.20859/supp-3 Supplemental Information 3 Permanent protective structures in lychee orchards and correct net installation practices Permanent protective structures in a lychee orchard: (A) with nets; (B) with chicken wire; (C) correct (left) and incorrect (right) net installation on an individual tree; and (D) white (left) and black (right) nets on trees.

10.7717/peerj.20859/supp-4 Supplemental Information 4 R script

10.7717/peerj.20859/supp-5 Supplemental Information 5 Tree-level estimates of fruit yield and fruit loss recorded as count data, attributed to animals and other factors in lychee and mango orchards in 2018, for unprotected trees and trees protected with a sound-light deterrent system

10.7717/peerj.20859/supp-6 Supplemental Information 6 Tree-level estimates of fruit yield and fruit loss recorded as count data, attributed to animals and other factors in a lychee orchard in 2023 and 2024, for trees protected with a netted structure and a structure with parallel cords

10.7717/peerj.20859/supp-7 Supplemental Information 7 Tree-level estimates of fruit yield and fruit loss recorded as count data, attributed to animals and other factors in lychee orchards in 2018, for unprotected trees and trees protected with odour-based repellents and flags

10.7717/peerj.20859/supp-8 Supplemental Information 8 Tree-level estimates of fruit yield and fruit loss recorded as count data, attributed to animals and other factors in a lychee orchard in 2021, for trees protected with a combined smoke-light deterrent and a netted structure

10.7717/peerj.20859/supp-9 Supplemental Information 9 Tree-level estimates of fruit yield and fruit loss recorded as count data, attributed to animals and other factors in a lychee orchard in 2024, for unprotected trees and trees protected with a sprinkler system

## Additional Information and Declarations

### Data Availability

The following information was supplied regarding data availability: The raw data are available in the Supplemental Files.
