# Supplementary material for: Fruitful outcomes without fatal costs: non-lethal alternatives show promise in alleviating human-wildlife conflict involving an island flying fox
Source: PeerJ. 2026 Mar 5;14:e20859. doi: 10.7717/peerj.20859 (PMC12967413; doi:10.7717/peerj.20859)
Supplement: Supplemental Information 1 — Additional details from the Methods section, including an overview of the sound-light system setup, the concentrations of odour-based repellents used, a figure showing the different non-lethal flying fox deterrent methods assessed, and a figure illustrating other types of fruit damage. [file peerj-14-20859-s001.docx]

**Appendix A. Supplementary methods information**


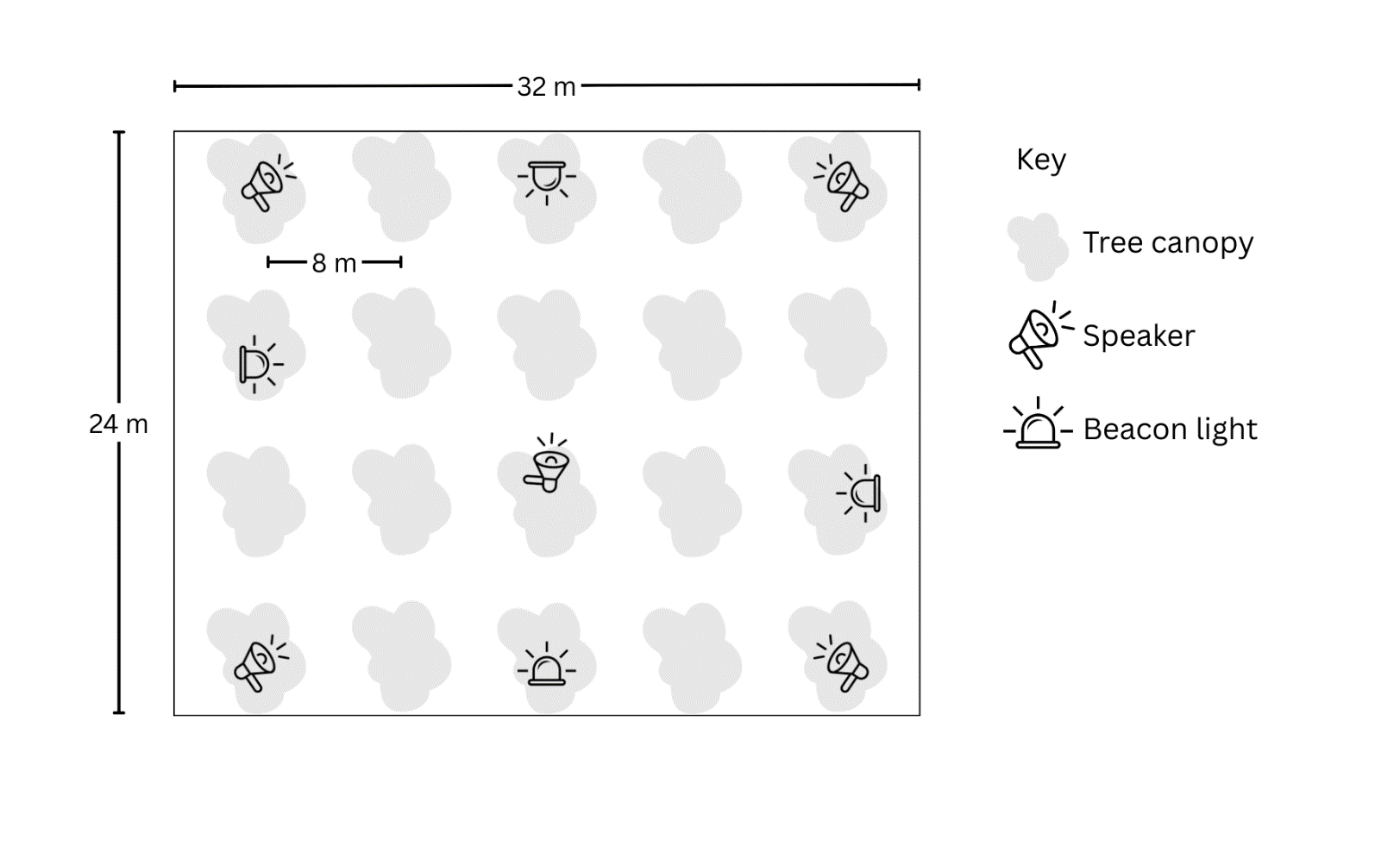


Figure A.1. Overview of the setup of the sound-light system in orchards with five speakers and four beacon lights, protecting 20 trees.

Table A.1. Main ingredients and concentration of odour-based repellents (diluted according to manufacturer’s instructions) used in lychee orchards in 2018.

| **Repellent ID** | **Brand name** | **Active ingredient** | **Ratio liquid concentrate:water** |
| --- | --- | --- | --- |
| Cr_1_ | I Must Garden® | Putrescent whole egg solid, garlic and mint oil (Imustgarden, 2025) | 1:9 |
| Cr_2_ | Repels-All® | Putrescent whole egg solid, clove, garlic, dried blood (Bonide, 2025) | 1:7 |
| Cr_3_ | Liquid Fence® | Putrescent whole egg solids, garlic and thyme oil (Liquid Fence, 2025) | 1:10 |
| Cr_4_ | Deer Off® | Putrescent egg, capsaicin and garlic (Havahart, 2025) | 1:7 |
| Hr | Homemade blend | Fresh rosemary, thyme, mint, black pepper and water | 50:25:10:10:1 |


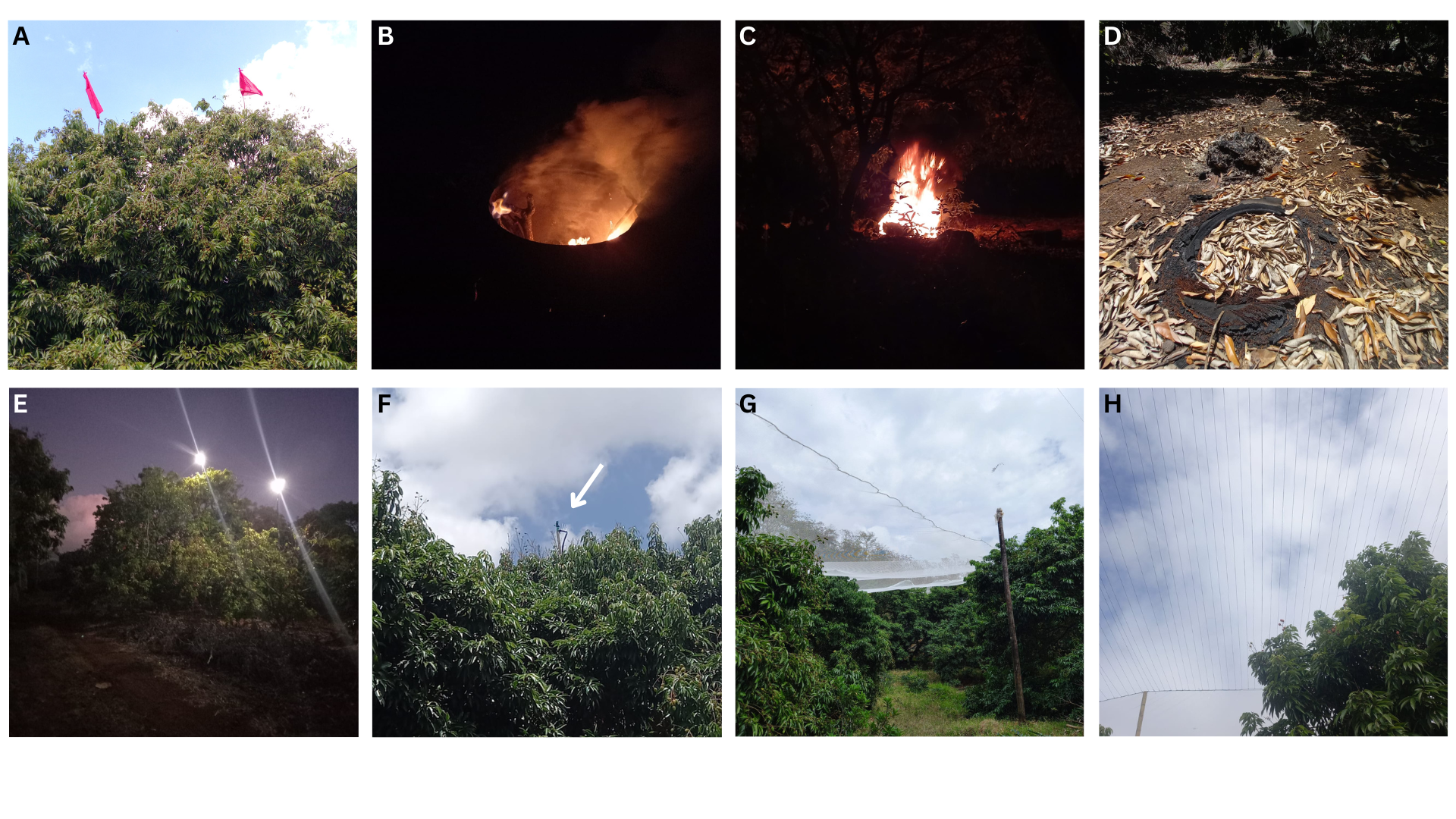


Figure A.2. Non-lethal methods assessed for protection of fruit trees from flying fox damage in Mauritius and tested in this study. (A) Flags soaked in repellent solution or without repellent. (B) Burning of dried lychee leaves and tyres (C, D) to create dense smoke at night. (E) Light bulbs above tree canopies. (F) Sprinkler (indicated by white arrow) above a tree canopy. (G) Lychee trees inside a fully netted structure. (H) Lace roofing above trees with nets on the sides.


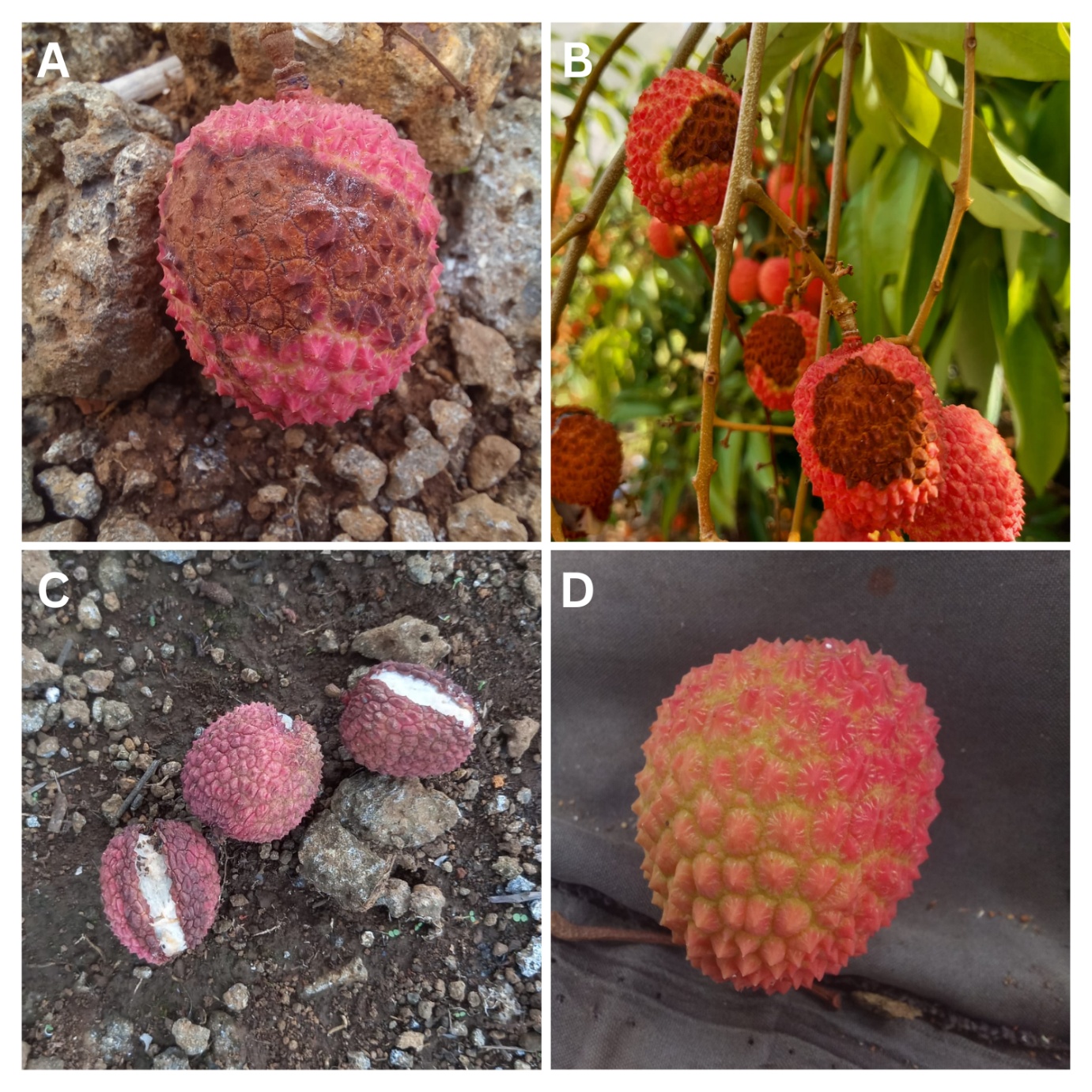


Figure A.3. Other types of damage recorded on lychee. (A) Sunburn (described in (Lal, Kumar & Pandey, 2023)), (B) disease (described in (Martínez-Bolaños et al., 2015), (C) fruit cracking and (D) natural fruit fall.

**References**

Bonide. 2025. Repels-All animal repellent concentrate. *Available at* *https://bonide.com/product/repels-all-animal-repellent-conc/* (accessed September 25, 2025).

Havahart. 2025. Deer Off deer repellent concentrate. *Available at* *https://www.domyown.com/deer-off-deer-repellent-concentrate-p-847.html* (accessed September 25, 2025).

Imustgarden. 2025. Deer Repellent - 1 gallon - mint scent. *Available at* *https://www.imustgarden.com/deer-repellent-mint-scent-1-gallon* (accessed September 25, 2025).

Lal N, Kumar A, Pandey SD. 2023. Sunburn and fruit cracking in Litchi (*Litchi chinensis* Sonn.) cv. ‘Rose Scented.’ *Emergent Life Sciences Research* 9:260–264. DOI: 10.31783/elsr.2023.92260264.

Liquid Fence. 2025. Deer & Rabbit Repellent Concentrate 2. *Available at* *https://www.liquidfence.com/products/deer-and-rabbit-control/deer-and-rabbit-concentrate.aspx* (accessed September 25, 2025).

Martínez-Bolaños M, Téliz-Ortiz D, Mora-Aguilera A, Valdovinos-Ponce G, Nieto-Ángel D, García-Pérez E, Sánchez-López V. 2015. Anthracnose (*Colletotrichum gloeosporioides* Penz.) of litchi fruit (*Litchi chinensis* Sonn.) in Oaxaca, México. *Revista Mexicana de Fitopatología* 33:140–155.
