# Supplementary figures and images for: Fruitful outcomes without fatal costs: non-lethal alternatives show promise in alleviating human-wildlife conflict involving an island flying fox
Source: PeerJ. 2026 Mar 5;14:e20859. doi: 10.7717/peerj.20859 (PMC12967413; doi:10.7717/peerj.20859)

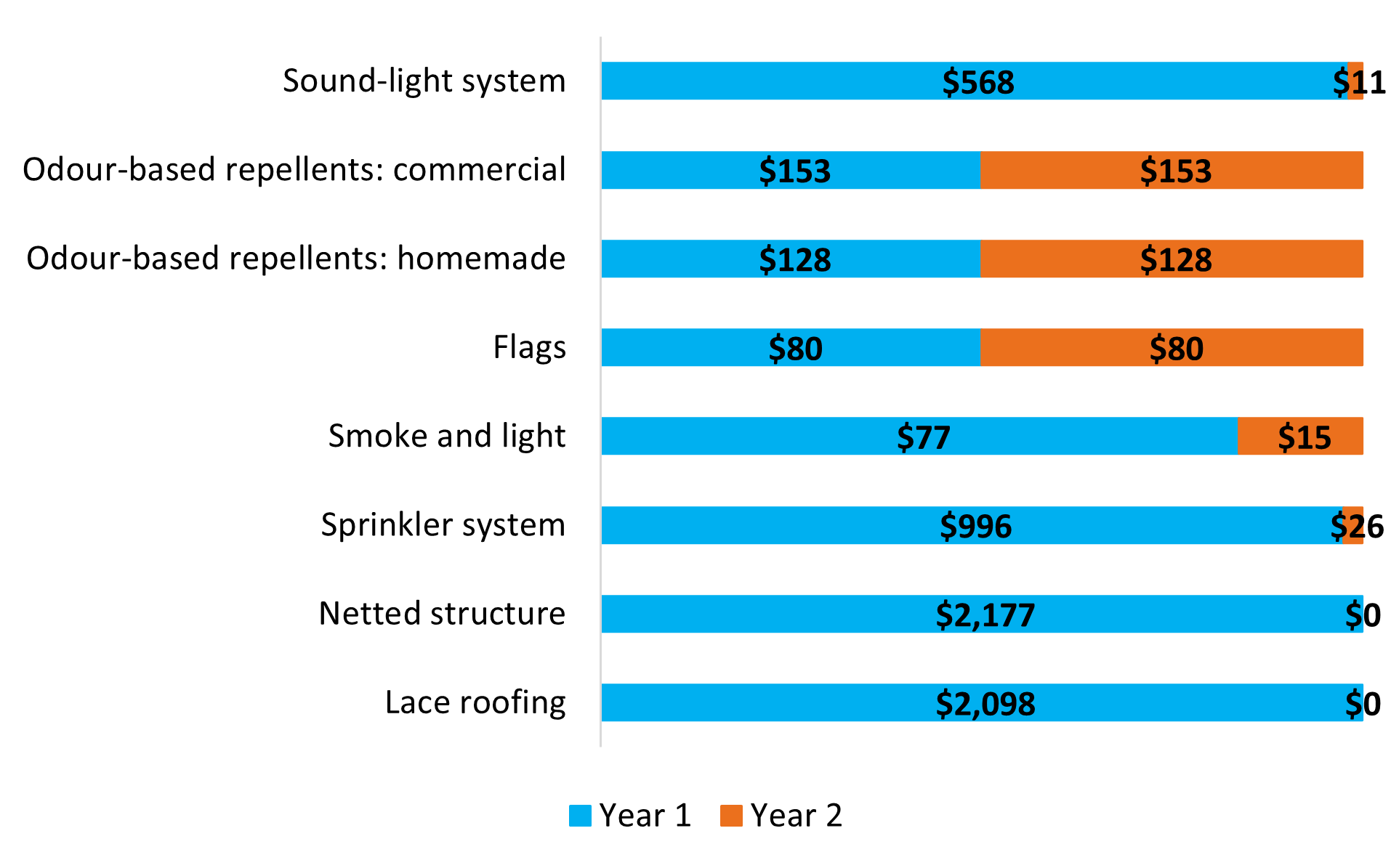

Supplement: Supplemental Information 2 — Investment cost of different flying fox deterrent methods tested during the study, shown for the first and second fruiting seasons (year 1 and year 2). The 100% stacked bar chart displays the proportionate cost of each deterrent type per year. Values on the chart represent estimated costs in USD (based on the exchange rate as of 29 April 2025) for 20 trees. The 100% stacked bar chart displays the proportionate cost of each deterrent type per year. Estimates include materials, equipment, and electricity and water supply, where applicable. Labour and software development costs are not included. [file peerj-14-20859-s002.png]

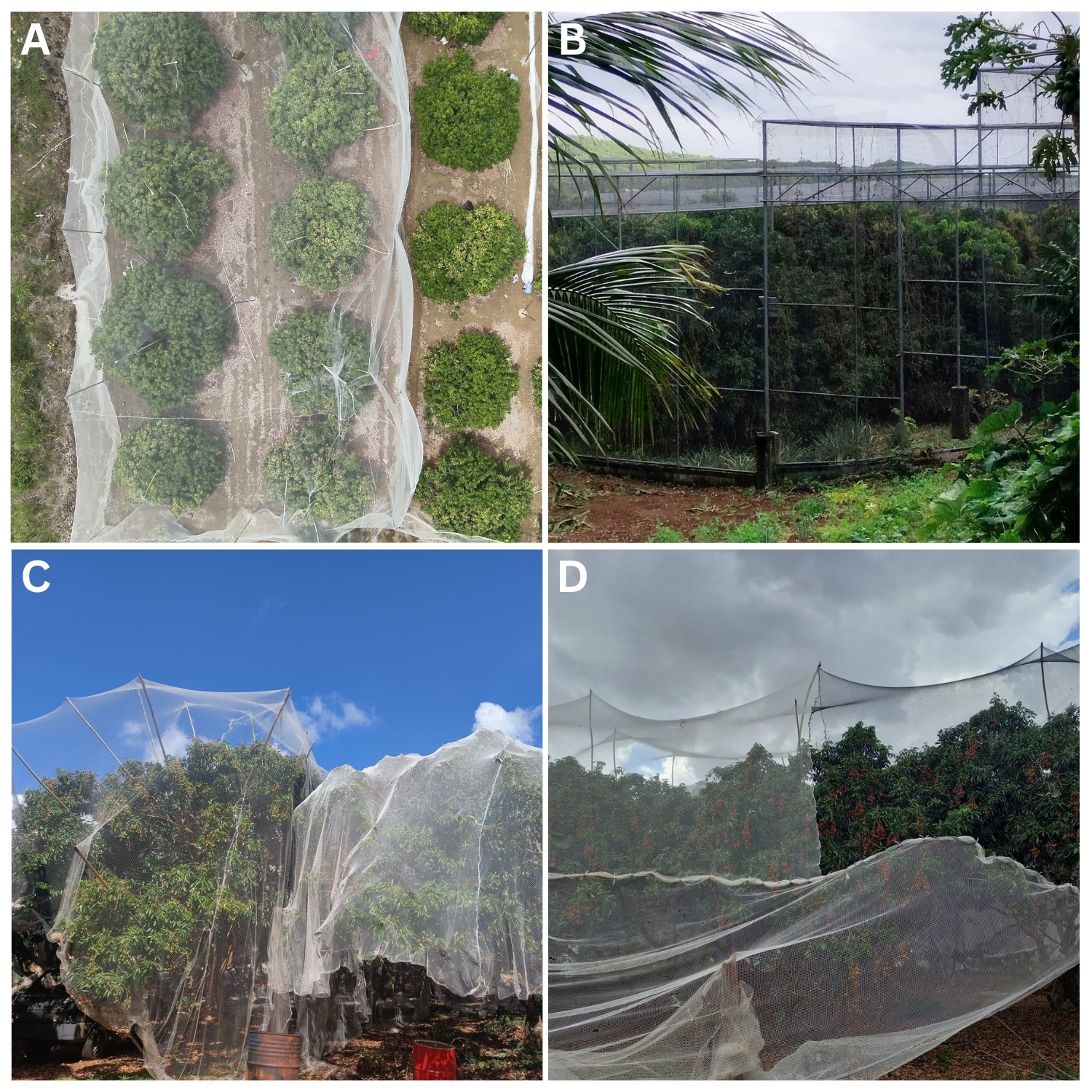

Supplement: Supplemental Information 3 — Permanent protective structures in a lychee orchard: (A) with nets; (B) with chicken wire; (C) correct (left) and incorrect (right) net installation on an individual tree; and (D) white (left) and black (right) nets on trees. [file peerj-14-20859-s003.png]
